# 3-Benzyl-6-isopropyl-5-phen­oxy-3*H*-1,2,3-triazolo[4,5-*d*]pyrimidin-7(6*H*)-one

**DOI:** 10.1107/S160053680904118X

**Published:** 2009-10-17

**Authors:** Shou-Heng Deng, Hong-Mei Wang, Ping Chen, Jun-Kai Ma, Feng-Jun Cao

**Affiliations:** a Center of Oncology, People’s Hospital affiliated with the YunYang Medical College, Shi Yan 442000, People’s Republic of China; b Institute of Medicinal Chemistry, Yunyang Medical College, Shiyan 442000, People’s Republic of China

## Abstract

In the title compound, C_20_H_19_N_5_O_2_, all atoms of the 1,2,3-triazolo[4,5-*d*]pyrimidine ring system are essentially coplanar [maximum deviation = 0.015 (2) Å], indicating the existence of a conjugate system in which each carbon and nitrogen atom is *sp* ^2^ hybridized and ten π electrons (three from carbon atoms and seven from nitrogen atoms) constitute an aromatic heterocycle. The ring system forms dihedral angles of 68.37 (10) and 71.57 (9)° with the phenyl rings. The crystal packing is stabilized by van der Waals inter­actions and intermolecular C—H⋯π interactions.

## Related literature

For the biological activity of 8-aza­guanine derivatives, see: Roblin *et al.* (1945 ▶); Ding *et al.* (2004 ▶); Mitchell *et al.* (1950 ▶); Levine *et al.* (1963 ▶); Montgomery *et al.* (1962 ▶); Yamamoto *et al.* (1967 ▶); Bariana (1971 ▶); Holland *et al.* (1975 ▶). For related structures, see: Ferguson *et al.* (1998 ▶); Li *et al.* (2004 ▶); Zhao, Xie *et al.* (2005 ▶); Zhao, Hu *et al.* (2005 ▶); Zhao, Wang & Ding (2005 ▶); Chen & Shi (2006 ▶); Maldonado *et al.* (2006 ▶); Xiao & Shi (2007 ▶); Wang *et al.* (2006 ▶, 2008 ▶); Zeng, Deng, Qu & Wang (2009 ▶); Zeng, Deng, Chen *et al.* (2009 ▶); Zeng, Liu *et al.* (2009 ▶).
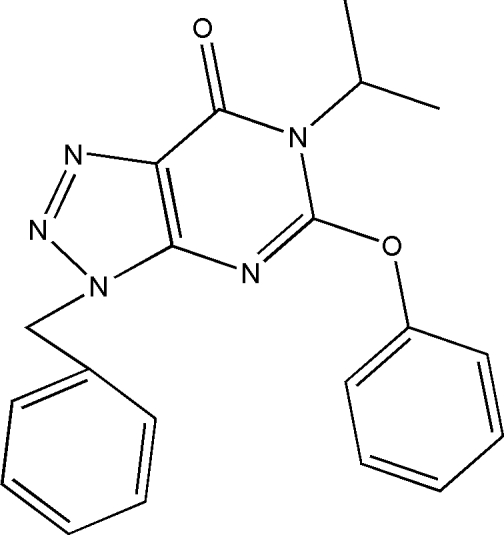


## Experimental

### 

#### Crystal data

C_20_H_19_N_5_O_2_
*M* *_r_* = 361.40
Monoclinic, 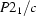

*a* = 9.4585 (13) Å
*b* = 9.0846 (12) Å
*c* = 21.992 (3) Å
β = 100.523 (2)°
*V* = 1858.0 (4) Å^3^
*Z* = 4
Mo *K*α radiation
μ = 0.09 mm^−1^
*T* = 298 K
0.16 × 0.13 × 0.10 mm

#### Data collection

Bruker SMART CCD area-detector diffractometer
Absorption correction: multi-scan (*SADABS*; Sheldrick, 1996 ▶) *T*_min_ = 0.986, *T*_max_ = 0.991
10907 measured reflections
3655 independent reflections
3079 reflections with *I* > 2σ(*I*)
*R*_int_ = 0.074

#### Refinement

*R*[*F* ^2^ > 2σ(*F* ^2^)] = 0.067
*wR*(*F* ^2^) = 0.167
*S* = 1.14
3655 reflections
246 parameters
H-atom parameters constrained
Δρ_max_ = 0.19 e Å^−3^
Δρ_min_ = −0.31 e Å^−3^

### 

Data collection: *SMART* (Bruker, 2001 ▶); cell refinement: *SAINT* (Bruker, 2001 ▶); data reduction: *SAINT*; program(s) used to solve structure: *SHELXS97* (Sheldrick, 2008 ▶); program(s) used to refine structure: *SHELXL97* (Sheldrick, 2008 ▶); molecular graphics: *PLATON* (Spek, 2009 ▶); software used to prepare material for publication: *SHELXTL* (Sheldrick, 2008 ▶).

## Supplementary Material

Crystal structure: contains datablocks global, I. DOI: 10.1107/S160053680904118X/rz2370sup1.cif

Structure factors: contains datablocks I. DOI: 10.1107/S160053680904118X/rz2370Isup2.hkl

Additional supplementary materials:  crystallographic information; 3D view; checkCIF report

## Figures and Tables

**Table 1:** Hydrogen-bond geometry (Å, °)

*D*—H⋯*A*	*D*—H	H⋯*A*	*D*⋯*A*	*D*—H⋯*A*
C13—H13*A*⋯*Cg*^i^	0.96	2.75	3.696 (3)	171

Symmetry code: (i) 
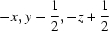
. *Cg* is the centroid of the C15–C20 phenyl ring.

**Fig. 1.**
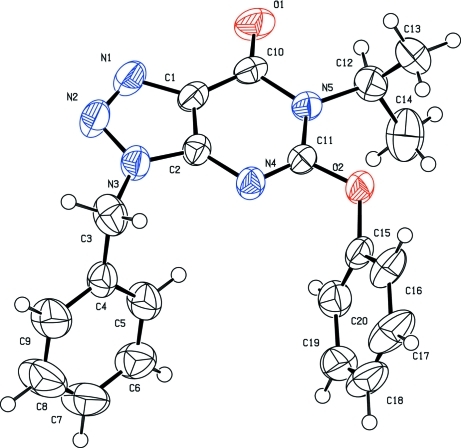
The molecular structure of the title compound showing the atom-labeling scheme. Displacement ellipsoids are drawn at 50% probability level. H-atoms are represented by circles of arbitrary size.

## supplementary crystallographic information

### Comment

The derivatives of heterocycles containing 8-azaguanine system, which are well known bioisosteres of guanine, are of great importance because of their remarkable biological properties. Some of these activities include antimicrobial or antifungal activities (Roblin *et al.*, 1945; Ding *et al.*, 2004), encephaloma cell inhibitor activity (Mitchell *et al.*, 1950; Levine *et al.*, 1963), antileukemie activity (Montgomery *et al.*, 1962), hypersusceptibility inhibitor activity and acesodyne activity (Yamamoto *et al.*, 1967; Bariana, 1971; Holland *et al.*, 1975).

In recent years, we have been engaged in the preparation of derivatives of 8-azaguanine *via* aza-Wittig reaction of beta-ethoxycarbonyl iminophosphorane with aromatic isocyanate (Zhao, Xie *et al.*, 2005). As a continuation of our research for new biologically active heterocycles, the title compound was obtained from beta-ethoxycarbonyl iminophosphorane with alphalic isocyanate, and structurally characterized in this context.

In the title compound (Fig. 1), bond lengths and angles within the triazolopyrimidinone moiety are in good agreement with those observed for closely related structures (Zhao, Hu *et al.*, 2005; Zhao, Wang & Ding, 2005). As reported for related compounds (Ferguson *et al.*, 1998; Maldonado *et al.*, 2006; Zeng, Deng, Qu *et al.*, 2009; Zeng, Deng, Chen *et al.*, 2009; Zeng, Liu *et al.*, 2009; Wang *et al.*, 2008; Xiao & Shi, 2007; Chen & Shi, 2007), all atoms in the 1,2,3-triazolo[4,5-*d*]pyrimidine ring system are essentially coplanar (maximum deviation 0.015 (2)Å for atom N2), indicating that the 1,2,3-triazolo[4,5-*d*]pyrimidine moiety is a conjugate system, in which each carbon and nitrogen atom is sp^2^ hybridized and ten π electrons (three from carbon atoms and seven from nitrogen atoms) constitute an aromatic heterocycle (Li *et al.*, 2004). The dihedral angles it forms with the C4–C9 and C15–C20 phenyl rings are 68.37 (10) and 71.57 (9)°, respectively.

The crystal packing is stabilized mainly by van der Waals interactions, no intermolecular hydrogen bonds or π–π stacking interactions being observed. One of the methyl H atoms is involved in a contact to the centroid (Cg) of the C15/C20 phenyl ring (C13–H13A···Cg = 2.75 Å), which may be considered as a C—H···π interaction.

### Experimental

To a solution of carbodiimide in CH_2_Cl_2_/CH_3_CN (1:4 *v*/*v*, 15 ml) prepared according to the literature method (Wang *et al.*, 2006), was added phenol (3 mmol) and excess K_2_CO_3_, and the reaction mixture was stirred for 12 h. The solvent was removed under reduced pressure and the residue was recrystallized from EtOH to give the title compound (yield 75%; m.p. 405 K). Elemental analysis: calculated for C_20_H_19_N_5_O_2_: C, 66.47; H, 5.30; N, 19.38%. Found: C, 65.67; H, 5.56; N, 18.92%. Crystals suitable for single-crystal X-ray diffraction analysis were obtained by slow evaporation of a hexane/dichloromethane (1:3 *v*/*v*) solution at room temperature.

### Refinement

H atoms were placed at calculated positions and treated as riding atoms, with C—H = 0.93–0.98 Å, and *U*_iso_(H) = 1.2*U*_eq_(C) or 1.5*U*_eq_(C) for methyl H atoms.

### Crystal data

**Table:**

C_20_H_19_N_5_O_2_	*F*(000) = 760
*M**_r_* = 361.40	*D*_x_ = 1.292 Mg m^−^^3^
Monoclinic, *P*2_1_/*c*	Mo *K*α radiation, λ = 0.71073 Å
Hall symbol: -P 2ybc	Cell parameters from 3682 reflections
*a* = 9.4585 (13) Å	θ = 2.4–25.1°
*b* = 9.0846 (12) Å	µ = 0.09 mm^−^^1^
*c* = 21.992 (3) Å	*T* = 298 K
β = 100.523 (2)°	Block, colourless
*V* = 1858.0 (4) Å^3^	0.16 × 0.13 × 0.10 mm
*Z* = 4	

### Data collection

**Table:**

Bruker SMART CCD area-detector diffractometer	3655 independent reflections
Radiation source: fine-focus sealed tube	3079 reflections with *I* > 2σ(*I*)
graphite	*R*_int_ = 0.074
φ and ω scans	θ_max_ = 26.0°, θ_min_ = 1.9°
Absorption correction: multi-scan (*SADABS*; Sheldrick, 1996)	*h* = −8→11
*T*_min_ = 0.986, *T*_max_ = 0.991	*k* = −11→11
10907 measured reflections	*l* = −27→27

### Refinement

**Table:**

Refinement on *F*^2^	Primary atom site location: structure-invariant direct methods
Least-squares matrix: full	Secondary atom site location: difference Fourier map
*R*[*F*^2^ > 2σ(*F*^2^)] = 0.067	Hydrogen site location: inferred from neighbouring sites
*wR*(*F*^2^) = 0.167	H-atom parameters constrained
*S* = 1.14	*w* = 1/[σ^2^(*F*_o_^2^) + (0.0685*P*)^2^ + 0.418*P*] where *P* = (*F*_o_^2^ + 2*F*_c_^2^)/3
3655 reflections	(Δ/σ)_max_ = 0.001
246 parameters	Δρ_max_ = 0.19 e Å^−^^3^
0 restraints	Δρ_min_ = −0.31 e Å^−^^3^

### Special details

**Table:**

Geometry. All e.s.d.'s (except the e.s.d. in the dihedral angle between two l.s. planes) are estimated using the full covariance matrix. The cell e.s.d.'s are taken into account individually in the estimation of e.s.d.'s in distances, angles and torsion angles; correlations between e.s.d.'s in cell parameters are only used when they are defined by crystal symmetry. An approximate (isotropic) treatment of cell e.s.d.'s is used for estimating e.s.d.'s involving l.s. planes.
Refinement. Refinement of *F*^2^ against ALL reflections. The weighted *R*-factor *wR* and goodness of fit *S* are based on *F*^2^, conventional *R*-factors *R* are based on *F*, with *F* set to zero for negative *F*^2^. The threshold expression of *F*^2^ > σ(*F*^2^) is used only for calculating *R*-factors(gt) *etc*. and is not relevant to the choice of reflections for refinement. *R*-factors based on *F*^2^ are statistically about twice as large as those based on *F*, and *R*- factors based on ALL data will be even larger.

### Fractional atomic coordinates and isotropic or equivalent isotropic displacement parameters (Å^2^)

**Table:**

	*x*	*y*	*z*	*U*_iso_*/*U*_eq_	
C1	0.3436 (2)	0.4731 (2)	0.22493 (9)	0.0422 (5)	
C2	0.3377 (2)	0.4926 (2)	0.28596 (9)	0.0400 (5)	
C3	0.4595 (3)	0.3594 (3)	0.38253 (11)	0.0615 (6)	
H3A	0.4907	0.2579	0.3884	0.074*	
H3B	0.3730	0.3701	0.4000	0.074*	
C4	0.5747 (2)	0.4573 (2)	0.41799 (10)	0.0486 (5)	
C5	0.5893 (3)	0.6038 (3)	0.40342 (11)	0.0616 (6)	
H5	0.5277	0.6448	0.3699	0.074*	
C6	0.6935 (3)	0.6893 (3)	0.43782 (12)	0.0697 (7)	
H6	0.7016	0.7878	0.4276	0.084*	
C7	0.7851 (4)	0.6317 (3)	0.48672 (12)	0.0810 (9)	
H7	0.8549	0.6906	0.5103	0.097*	
C8	0.7738 (4)	0.4858 (4)	0.50106 (13)	0.0974 (11)	
H8	0.8373	0.4450	0.5340	0.117*	
C9	0.6685 (3)	0.3994 (3)	0.46668 (12)	0.0751 (8)	
H9	0.6614	0.3006	0.4768	0.090*	
C10	0.2657 (2)	0.5691 (2)	0.17917 (9)	0.0435 (5)	
C11	0.1931 (2)	0.6823 (2)	0.26940 (9)	0.0409 (5)	
C12	0.0995 (3)	0.7849 (3)	0.16396 (10)	0.0594 (6)	
H12	0.1186	0.7635	0.1225	0.071*	
C13	−0.0597 (3)	0.7597 (3)	0.16099 (13)	0.0810 (9)	
H13A	−0.0837	0.6606	0.1478	0.121*	
H13B	−0.1133	0.8274	0.1321	0.121*	
H13C	−0.0831	0.7751	0.2012	0.121*	
C14	0.1452 (4)	0.9438 (3)	0.17780 (15)	0.0853 (9)	
H14A	0.1002	0.9807	0.2105	0.128*	
H14B	0.1163	1.0025	0.1414	0.128*	
H14C	0.2478	0.9483	0.1903	0.128*	
C15	0.1523 (3)	0.8427 (2)	0.34770 (9)	0.0479 (5)	
C16	0.0476 (3)	0.8378 (3)	0.38311 (12)	0.0635 (7)	
H16	−0.0422	0.7977	0.3677	0.076*	
C17	0.0788 (4)	0.8938 (3)	0.44193 (13)	0.0838 (10)	
H17	0.0090	0.8917	0.4667	0.101*	
C18	0.2099 (4)	0.9524 (3)	0.46459 (11)	0.0810 (10)	
H18	0.2297	0.9888	0.5048	0.097*	
C19	0.3141 (3)	0.9579 (3)	0.42792 (12)	0.0723 (8)	
H19	0.4035	0.9991	0.4432	0.087*	
C20	0.2849 (3)	0.9021 (3)	0.36845 (10)	0.0591 (6)	
H20	0.3538	0.9049	0.3432	0.071*	
N1	0.4331 (2)	0.3583 (2)	0.21950 (10)	0.0601 (5)	
N2	0.4819 (2)	0.3075 (2)	0.27476 (11)	0.0633 (6)	
N3	0.42351 (19)	0.38878 (19)	0.31642 (8)	0.0488 (5)	
N4	0.26237 (18)	0.59583 (19)	0.31105 (7)	0.0444 (4)	
N5	0.18677 (18)	0.67677 (18)	0.20678 (7)	0.0411 (4)	
O1	0.26306 (19)	0.56707 (19)	0.12390 (7)	0.0635 (5)	
O2	0.11490 (17)	0.79269 (18)	0.28643 (6)	0.0572 (5)	

### Atomic displacement parameters (Å^2^)

**Table:**

	*U*^11^	*U*^22^	*U*^33^	*U*^12^	*U*^13^	*U*^23^
C1	0.0411 (11)	0.0384 (10)	0.0493 (11)	−0.0012 (8)	0.0138 (9)	−0.0112 (8)
C2	0.0374 (10)	0.0343 (9)	0.0487 (11)	0.0003 (8)	0.0094 (9)	−0.0007 (8)
C3	0.0607 (15)	0.0502 (13)	0.0711 (16)	0.0003 (11)	0.0051 (12)	0.0202 (11)
C4	0.0540 (13)	0.0454 (11)	0.0475 (12)	0.0092 (10)	0.0123 (10)	0.0075 (9)
C5	0.0682 (16)	0.0488 (13)	0.0632 (15)	0.0028 (12)	0.0000 (12)	0.0114 (11)
C6	0.091 (2)	0.0526 (14)	0.0628 (16)	−0.0057 (14)	0.0072 (14)	0.0002 (11)
C7	0.106 (2)	0.0769 (19)	0.0526 (15)	−0.0154 (17)	−0.0042 (15)	−0.0061 (13)
C8	0.125 (3)	0.089 (2)	0.0612 (17)	−0.006 (2)	−0.0294 (18)	0.0158 (15)
C9	0.100 (2)	0.0570 (15)	0.0616 (15)	0.0009 (15)	−0.0032 (15)	0.0169 (12)
C10	0.0478 (12)	0.0438 (11)	0.0403 (11)	−0.0039 (9)	0.0115 (9)	−0.0117 (8)
C11	0.0415 (11)	0.0433 (11)	0.0387 (10)	0.0057 (9)	0.0089 (8)	−0.0045 (8)
C12	0.0767 (17)	0.0649 (15)	0.0354 (11)	0.0199 (13)	0.0073 (11)	0.0058 (10)
C13	0.0683 (18)	0.0792 (18)	0.0812 (19)	0.0162 (15)	−0.0237 (15)	−0.0102 (15)
C14	0.095 (2)	0.0628 (17)	0.103 (2)	0.0139 (16)	0.0296 (18)	0.0320 (16)
C15	0.0629 (14)	0.0431 (11)	0.0394 (11)	0.0186 (10)	0.0133 (10)	−0.0029 (8)
C16	0.0803 (18)	0.0497 (13)	0.0690 (15)	0.0098 (12)	0.0361 (14)	−0.0083 (11)
C17	0.137 (3)	0.0626 (17)	0.0659 (18)	0.0074 (19)	0.0572 (19)	−0.0088 (14)
C18	0.152 (3)	0.0538 (15)	0.0372 (12)	0.0233 (18)	0.0171 (17)	−0.0061 (11)
C19	0.093 (2)	0.0560 (15)	0.0598 (15)	0.0115 (14)	−0.0083 (14)	−0.0043 (12)
C20	0.0656 (16)	0.0636 (15)	0.0489 (13)	0.0154 (13)	0.0126 (11)	0.0025 (11)
N1	0.0563 (12)	0.0541 (11)	0.0707 (13)	0.0107 (9)	0.0138 (10)	−0.0194 (10)
N2	0.0584 (13)	0.0452 (11)	0.0843 (15)	0.0133 (9)	0.0081 (11)	−0.0134 (10)
N3	0.0466 (10)	0.0381 (9)	0.0604 (11)	0.0049 (8)	0.0063 (8)	0.0010 (8)
N4	0.0486 (10)	0.0474 (10)	0.0387 (9)	0.0117 (8)	0.0121 (7)	0.0014 (7)
N5	0.0456 (10)	0.0428 (9)	0.0350 (8)	0.0036 (7)	0.0079 (7)	−0.0024 (7)
O1	0.0826 (12)	0.0719 (11)	0.0383 (8)	0.0049 (9)	0.0172 (8)	−0.0126 (7)
O2	0.0638 (10)	0.0664 (10)	0.0401 (8)	0.0306 (8)	0.0057 (7)	−0.0096 (7)

### Geometric parameters (Å, °)

**Table:**

C1—N1	1.362 (3)	C12—N5	1.500 (3)
C1—C2	1.365 (3)	C12—C13	1.513 (4)
C1—C10	1.430 (3)	C12—C14	1.521 (4)
C2—N3	1.341 (2)	C12—H12	0.9800
C2—N4	1.355 (2)	C13—H13A	0.9600
C3—N3	1.456 (3)	C13—H13B	0.9600
C3—C4	1.509 (3)	C13—H13C	0.9600
C3—H3A	0.9700	C14—H14A	0.9600
C3—H3B	0.9700	C14—H14B	0.9600
C4—C9	1.365 (3)	C14—H14C	0.9600
C4—C5	1.382 (3)	C15—C20	1.365 (3)
C5—C6	1.369 (3)	C15—C16	1.367 (3)
C5—H5	0.9300	C15—O2	1.405 (2)
C6—C7	1.356 (4)	C16—C17	1.371 (4)
C6—H6	0.9300	C16—H16	0.9300
C7—C8	1.371 (4)	C17—C18	1.358 (5)
C7—H7	0.9300	C17—H17	0.9300
C8—C9	1.380 (4)	C18—C19	1.383 (4)
C8—H8	0.9300	C18—H18	0.9300
C9—H9	0.9300	C19—C20	1.383 (3)
C10—O1	1.211 (2)	C19—H19	0.9300
C10—N5	1.431 (2)	C20—H20	0.9300
C11—N4	1.291 (2)	N1—N2	1.303 (3)
C11—O2	1.339 (2)	N2—N3	1.369 (3)
C11—N5	1.368 (2)		
			
N1—C1—C2	108.83 (19)	C14—C12—H12	106.3
N1—C1—C10	130.83 (19)	C12—C13—H13A	109.5
C2—C1—C10	120.31 (18)	C12—C13—H13B	109.5
N3—C2—N4	126.77 (18)	H13A—C13—H13B	109.5
N3—C2—C1	105.72 (17)	C12—C13—H13C	109.5
N4—C2—C1	127.51 (18)	H13A—C13—H13C	109.5
N3—C3—C4	115.02 (18)	H13B—C13—H13C	109.5
N3—C3—H3A	108.5	C12—C14—H14A	109.5
C4—C3—H3A	108.5	C12—C14—H14B	109.5
N3—C3—H3B	108.5	H14A—C14—H14B	109.5
C4—C3—H3B	108.5	C12—C14—H14C	109.5
H3A—C3—H3B	107.5	H14A—C14—H14C	109.5
C9—C4—C5	118.3 (2)	H14B—C14—H14C	109.5
C9—C4—C3	118.9 (2)	C20—C15—C16	122.6 (2)
C5—C4—C3	122.7 (2)	C20—C15—O2	120.2 (2)
C6—C5—C4	120.7 (2)	C16—C15—O2	117.0 (2)
C6—C5—H5	119.7	C15—C16—C17	118.2 (3)
C4—C5—H5	119.7	C15—C16—H16	120.9
C7—C6—C5	120.7 (2)	C17—C16—H16	120.9
C7—C6—H6	119.6	C18—C17—C16	121.1 (3)
C5—C6—H6	119.6	C18—C17—H17	119.5
C6—C7—C8	119.3 (3)	C16—C17—H17	119.5
C6—C7—H7	120.3	C17—C18—C19	120.0 (2)
C8—C7—H7	120.3	C17—C18—H18	120.0
C7—C8—C9	120.2 (3)	C19—C18—H18	120.0
C7—C8—H8	119.9	C20—C19—C18	119.8 (3)
C9—C8—H8	119.9	C20—C19—H19	120.1
C4—C9—C8	120.8 (2)	C18—C19—H19	120.1
C4—C9—H9	119.6	C15—C20—C19	118.3 (2)
C8—C9—H9	119.6	C15—C20—H20	120.9
O1—C10—C1	127.61 (19)	C19—C20—H20	120.9
O1—C10—N5	121.29 (19)	N2—N1—C1	108.03 (18)
C1—C10—N5	111.10 (16)	N1—N2—N3	108.40 (17)
N4—C11—O2	119.46 (17)	C2—N3—N2	109.02 (18)
N4—C11—N5	127.95 (18)	C2—N3—C3	129.62 (19)
O2—C11—N5	112.58 (16)	N2—N3—C3	121.34 (19)
N5—C12—C13	110.9 (2)	C11—N4—C2	111.65 (16)
N5—C12—C14	113.1 (2)	C11—N5—C10	121.45 (16)
C13—C12—C14	113.3 (2)	C11—N5—C12	121.72 (16)
N5—C12—H12	106.3	C10—N5—C12	116.81 (16)
C13—C12—H12	106.3	C11—O2—C15	117.06 (15)
			
N1—C1—C2—N3	−0.2 (2)	N4—C2—N3—N2	−179.02 (19)
C10—C1—C2—N3	−178.61 (18)	C1—C2—N3—N2	0.4 (2)
N1—C1—C2—N4	179.23 (19)	N4—C2—N3—C3	−0.5 (3)
C10—C1—C2—N4	0.8 (3)	C1—C2—N3—C3	178.9 (2)
N3—C3—C4—C9	−145.0 (2)	N1—N2—N3—C2	−0.5 (2)
N3—C3—C4—C5	35.3 (3)	N1—N2—N3—C3	−179.14 (19)
C9—C4—C5—C6	−1.3 (4)	C4—C3—N3—C2	−80.8 (3)
C3—C4—C5—C6	178.5 (2)	C4—C3—N3—N2	97.6 (2)
C4—C5—C6—C7	0.3 (4)	O2—C11—N4—C2	−178.81 (18)
C5—C6—C7—C8	0.9 (5)	N5—C11—N4—C2	1.8 (3)
C6—C7—C8—C9	−1.2 (6)	N3—C2—N4—C11	178.18 (19)
C5—C4—C9—C8	1.0 (4)	C1—C2—N4—C11	−1.1 (3)
C3—C4—C9—C8	−178.8 (3)	N4—C11—N5—C10	−2.1 (3)
C7—C8—C9—C4	0.2 (5)	O2—C11—N5—C10	178.45 (17)
N1—C1—C10—O1	0.3 (4)	N4—C11—N5—C12	179.2 (2)
C2—C1—C10—O1	178.4 (2)	O2—C11—N5—C12	−0.2 (3)
N1—C1—C10—N5	−178.9 (2)	O1—C10—N5—C11	−177.88 (19)
C2—C1—C10—N5	−0.8 (3)	C1—C10—N5—C11	1.4 (3)
C20—C15—C16—C17	−0.7 (4)	O1—C10—N5—C12	0.9 (3)
O2—C15—C16—C17	−176.3 (2)	C1—C10—N5—C12	−179.87 (18)
C15—C16—C17—C18	−0.1 (4)	C13—C12—N5—C11	−69.5 (3)
C16—C17—C18—C19	0.8 (4)	C14—C12—N5—C11	59.1 (3)
C17—C18—C19—C20	−0.8 (4)	C13—C12—N5—C10	111.8 (2)
C16—C15—C20—C19	0.7 (3)	C14—C12—N5—C10	−119.6 (2)
O2—C15—C20—C19	176.16 (19)	N4—C11—O2—C15	22.9 (3)
C18—C19—C20—C15	0.1 (4)	N5—C11—O2—C15	−157.54 (18)
C2—C1—N1—N2	−0.1 (2)	C20—C15—O2—C11	62.1 (3)
C10—C1—N1—N2	178.1 (2)	C16—C15—O2—C11	−122.2 (2)
C1—N1—N2—N3	0.3 (2)		

### Hydrogen-bond geometry (Å, °)

**Table:**

*D*—H···*A*	*D*—H	H···*A*	*D*···*A*	*D*—H···*A*
C13—H13A···Cg^i^	0.96	2.75	3.696 (3)	171

Symmetry codes: (i) −*x*, *y*−1/2, −*z*+1/2.

## References

[bb1] Bariana, D. S. (1971). *J. Med. Chem.* **14**, 535–543. 10.1021/jm00288a020 5091971

[bb2] Bruker (2001). *SMART* and *SAINT* Bruker AXS Inc., Madison, Wisconsin, USA.

[bb3] Chen, X.-B. & Shi, D.-Q. (2006). *Acta Cryst.* E**62**, o4780–o4782.

[bb4] Ding, M. W., Xu, S. Z. & Zhao, J. F. (2004). *J. Org. Chem.* **69**, 8366–8371. 10.1021/jo048691v 15549808

[bb5] Ferguson, G., Low, J. N., Nogueras, M., Cobo, J., Lopez, M. D., Quijano, M. L. & Sanchez, A. (1998). *Acta Cryst.* C**54**, IUC9800031.

[bb6] Holland, A., Jackson, D., Chaplen, P., Lunt, E., Marshall, S., Pain, C. L. & Wooldridge, K. R. H. (1975). *Eur. J. Med. Chem.* **10**, 447–449.

[bb7] Levine, R. J., Hall, T. C. & Harris, C. A. (1963). *Cancer (N. Y.)*, **16**, 269–272. 10.1002/1097-0142(196302)16:2<269::aid-cncr2820160218>3.0.co;2-v 13930160

[bb8] Li, M., Wen, L. R., Fu, W. J., Hu, F. Z. & Yang, H. Z. (2004). *Chin. J. Struct. Chem.* **23**, 11–14.

[bb9] Maldonado, C. R., Quirós, M. & Salas, J. M. (2006). *Acta Cryst.* C**62**, o489–o491. 10.1107/S0108270106022359 16891728

[bb10] Mitchell, J. H., Skipper, H. E. & Bennett, L. L. (1950). *Cancer Res.* **10**, 647–649. 15434808

[bb11] Montgomery, J. A., Schabel, F. M. & Skipper, H. E. (1962). *Cancer Res.* **22**, 504–509. 14475543

[bb12] Roblin, R. O., Lampen, J. O., English, J. P., Cole, Q. P. & Vaughan, J. R. (1945). *J. Am. Chem. Soc.* **67**, 290–294.

[bb13] Sheldrick, G. M. (1996). *SADABS* University of Göttingen, Germany.

[bb14] Sheldrick, G. M. (2008). *Acta Cryst.* A**64**, 112–122. 10.1107/S0108767307043930 18156677

[bb15] Spek, A. L. (2009). *Acta Cryst.* D**65**, 148–155. 10.1107/S090744490804362X PMC2631630 19171970

[bb16] Wang, H.-M., Chen, L.-L., Hu, T. & Zeng, X.-H. (2008). *Acta Cryst.* E**64**, o2404. 10.1107/S160053680803732X PMC2960130 21581373

[bb17] Wang, H.-M., Zeng, X.-H., Hu, Z.-Q., Li, G.-H. & Tian, J.-H. (2006). *Acta Cryst.* E**62**, o5038–o5040.

[bb18] Xiao, L.-X. & Shi, D.-Q. (2007). *Acta Cryst.* E**63**, o2843.

[bb19] Yamamoto, I., Inoki, R., Tamari, Y. & Iwatsubo, K. (1967). *Jpn J. Pharmacol.* **17**, 140–142. 10.1254/jjp.17.140 5299971

[bb20] Zeng, X.-H., Deng, S.-H., Chen, P., Wang, H.-M. & Gao, H.-T. (2009). *Acta Cryst.* E**65**, o2653–o2654. 10.1107/S1600536809039798 PMC2971036 21578267

[bb21] Zeng, X.-H., Deng, S.-H., Qu, Y.-N. & Wang, H.-M. (2009). *Acta Cryst.* E**65**, o1142–o1143. 10.1107/S1600536809014962 PMC2977812 21583949

[bb22] Zeng, X.-H., Liu, X.-L., Deng, S.-H., Chen, P. & Wang, H.-M. (2009). *Acta Cryst.* E**65**, o2583–o2584. 10.1107/S160053680903788X PMC2970466 21578020

[bb23] Zhao, J.-F., Hu, Y.-G., Ding, M.-W. & He, H.-W. (2005). *Acta Cryst.* E**61**, o2791–o2792.

[bb24] Zhao, J. F., Wang, C. G. & Ding, M. W. (2005). *Chin. J. Struct. Chem.* **24**, 439–444.

[bb25] Zhao, J. F., Xie, C., Ding, M. W. & He, H. W. (2005). *Chem. Lett.* **34**, 1020–1022.

